# *Xianling Lianxia* formula enhances the inhibitory effects of trastuzumab on HER2-positive breast cancer: Effect of *Xianling Lianxia* formula on HER2-positive breast cancer

**DOI:** 10.3724/abbs.2023281

**Published:** 2024-02-20

**Authors:** Feifei Li, Yuanyuan Wu, Youyang Shi, Xiaofei Liu, Ying Xie, Sheng Liu

**Affiliations:** 1 Department of Breast Surgery Longhua Hospital Shanghai University of Traditional Chinese Medicine Shanghai 200030 China; 2 Department of Thyroid and Breast Surgery Affiliated Hospital of Shandong University of Traditional Chinese Medicine Jinan 250014 China; 3 Yueyang Hospital of Integrated Chinese and Western Medicine Shanghai University of Traditional Chinese Medicine Shanghai 200437 China

**Keywords:** HER2-positive breast cancer, trastuzumab, invasive and metastatic, inflammation factor

## Abstract

Human epidermal growth factor receptor 2 (HER2)-positive breast cancer (BC) is characterized by high invasiveness. Trastuzumab considerably improves the prognoses of HER2-positive BC, but some patients exhibit drug resistance. In this study, the effects of XLLXF combined with trastuzumab on the proliferation, apoptosis, invasion, and migration of HER2-positive BC cells are evaluated, and network pharmacology is performed. Then, we conduct an *in vivo* study using a xenograft mouse model of HER2-positive BC, and tumor growth is monitored. The expression levels of cytokines are measured by ELISA. Molecular docking is performed to observe the binding stability of IL2, JAK, STAT, and TNF with curcumenol, icariside-II, lobetyolin, and scutellarein. Finally, we observe changes in JAK1 and TNF-α in tumor tissues by immunohistochemistry. The results show that XLLXF enhances the inhibitory effects of trastuzumab on the proliferation, colony formation ability, migration, and invasion of HER2-positive BC cells and promotes apoptosis. Network pharmacology reveals that XLLXF may exert its effects on HER2-positive BC by modulating pathways such as the ErbB, JAK-STAT, and NF-κB pathways. Potential targets include cytokines closely related to immune function. In the *in vivo* study, XLLXF synergistically enhances the inhibitory effects of trastuzumab on tumor growth. ELISA reveals that XLLXF combined with trastuzumab increases the levels of IL-15, IL-2, TNF-α, and IFN-γ in tumor-bearing mice. Immunohistochemistry confirms that XLLXF can regulate the expressions of JAK1 and TNF-α. This study demonstrates that XLLXF can synergistically enhance the efficacy of trastuzumab in targeting HER2-positive BC. The mechanism may involve the modulation of inflammatory factors.

## Introduction

Breast cancer (BC) is the most common cancer in women worldwide and the second leading cause of cancer-related deaths. It is the top malignant tumor in terms of incidence in women, and human epidermal growth factor receptor 2 (HER2)-positive BC accounts for approximately 15%–25% of cases. This subtype is characterized by its aggressive nature and poor prognosis [1]. Trastuzumab exerts its inhibitory effects on HER2-positive BC through various mechanisms [2], including blocking the activation of homodimers and triggering immune cells to kill cancer cells [3]. However, although it considerably improves the clinical outcomes of patients with HER2-positive BC, its clinical benefits are inevitably limited by inherent or acquired resistance [4].

Traditional Chinese medicine (TCM) treatment possesses unique benefits owing to its multicomponent and multitarget characteristics, showing significant efficacy in inhibiting tumor proliferation, invasion, and metastasis and synergistic enhancement [ 5, 6]. With more than 20 years of clinical experience, our team formulated the TCM prescription XLLXF for the treatment of BC. We found that XLLXF inhibits invasion and metastasis of triple-negative BC [7] and improves the 3-year disease-free survival (DFS) of patients with HER2-positive BC [8].

Based on these findings, we conducted CCK-8 assay, colony formation assay, Transwell assay, wound healing assay, network pharmacology study, and *in vivo* experiments to explore an effective TCM clinical treatment regimen for preventing the recurrence and metastasis of HER2-positive BC. It holds significant importance for the treatment of patients with HER2-positive BC.

## Materials and Methods

### Cell lines and cell culture

The human HER2-positive BC cell lines SK-BR-3 and JIMT-1 (Cell Bank of the Chinese Academy of Sciences, Shanghai, China) were selected for the experiments. They were tested for mycoplasma and cultured in DMEM (Gibco, Carlsbad, USA) supplemented with 10% FBS (Gibco). The cells were incubated at 37°C in a 5% CO _2_ incubator with saturated humidity.

### Experimental drugs

XLLXF was composed of Epimedii Folium ( *Epimedium brevicornum* Maxim., abbreviation: XLP), Codonopsis Radix [ *Codonopsis pilosula* (Franch.) Nannf., abbreviation: DS], Poria [ *Poria cocos* (Schw.) Wolf, abbreviation: FL], Curcumae Rhizoma ( *Curcuma aeruginosa Roxb*.C.zedoaria non Rosc., abbreviation: EZ), Scutellaria Barbata [ *Scutellaria barbata D* . *Don* (S. rivularis Wall.), abbreviation: BZL], and Prunellae Spica ( *Prunella vulgaris* L., abbreviation: XKC). The following quantities of each herb were used: 15 g of XLP, 12 g of DS, 12 g of FL, 30 g of BZL, 30 g of EZ, and 9 g of XKC. The herbs were placed in a decoction pot and soaked in 1080 mL of distilled water for 1 h. After boiling for 1 h, the herbal liquid was collected by filtering through gauze. Another 1080 mL of water was added, the mixture was boiled for 1 h, and the liquid was collected again by filtering. The two herbal liquids were mixed thoroughly, concentrated with a rotary evaporator, and freeze-dried in a freeze dryer for 72 h to obtain the freeze-dried powder of XLLXF. The yield of XLLXF was 29.23% (extract weight/herb weight).

Trastuzumab (manufacturer: Roche Pharmaceuticals Ltd., Shanghai, China; trade name: Herceptin; batch number: S20110020; specification: 440 mg; 20 mL/bottle) was purchased from the Western Medicine Pharmacy of Longhua Hospital Affiliated to the Shanghai University of Traditional Chinese Medicine. Lyophilized trastuzumab powder (440 mg) was dissolved in 20 mL of the provided diluent. The solution had a concentration of 21 mg/mL and a pH of approximately 6.0.

### CCK8 assay

SK-BR-3 and JIMT-1 cells in the logarithmic growth phase and in good condition were seeded in 96-well plates at a volume of 100 μL (8×10 ^4^ cells/mL) per well. After the cells adhered to the plate, the medium was replaced by fresh culture medium containing different concentrations of XLLXF (0, 6.25, 12.5, 50, 100, 200, and 400 μg/mL), trastuzumab (0, 0.04, 0.08, 0.17, 0.34, 0.68, 1.35, 2.70, 5.41, and 10.81 μM), or their combinations. The effects of the drugs on cell proliferation were investigated by cell counting kit-8 (CCK8) assay. Briefly, after 24 or 48 h of drug intervention, 10 μL of CCK8 solution (Beyotime, Shanghai, China) was added to each well, and the cells were incubated in a cell culture incubator for 2 h. The 96-well plate was then placed in a microplate reader (Berten Instruments, Shanghai, China), and the absorbance of each well was measured at 450 nm.

### Colony formation assay

The experimental groups included a blank control group, trastuzumab group (2.7 μM), XLLXF group (100 μg/mL), and trastuzumab+XLLXF group. When the cells reached the logarithmic growth phase, SK-BR-3 and JIMT-1 cells were harvested, and after digestion, the cells were seeded into 6-well plates at a density of approximately 1000–2000 cells per well (2 mL per well). The plates were then placed in an incubator for cultivation. The culture medium was replaced regularly, and after approximately 2 weeks, the colonies were observed and photographed under an inverted microscope (Olympus, Tokyo, Japan). The effects of the drugs on colony formation ability were analyzed.

### Cell apoptosis assay

SK-BR-3 and JIMT-1 cells were harvested and seeded into 6-well plates at a density of 2 mL per well. The plates were placed in an incubator (37°C with 5% CO _2_). After the cells reached 80%–90% confluence, drug intervention was performed. After 24 or 48 h of drug intervention, the cells were collected, washed with PBS, and resuspended in 1× binding buffer (Biolegend, San Diego, USA). The cells were then centrifuged at 4°C and 300 *g* for 5 min, and the process was repeated twice. The cells were diluted to a concentration of 1×10 ^6^ cells/mL with 1× binding buffer. Then, 100 μL of the cell suspension was transferred to 1.5 mL EP tubes, and 5 μL of FITC annexin V (Biolegend) and propidium iodide were added to each tube. The tubes were incubated at room temperature in the dark for 15 min. After incubation, the cells were centrifuged at 4°C and 300 *g* for 5 min, and the supernatant was discarded. The cells were resuspended in 200 μL of 1× binding buffer. Flow cytometry was then used to detect cell apoptosis.

### JC-1 staining for mitochondrial membrane potential

After 24 h of drug intervention, the cells of each group were treated with 1 mL of JC-1 staining working solution (Beyotime) and incubated at 37°C with 5% CO _2_ for 20 min. During the incubation, JC-1 staining buffer (1×) was prepared by dilution with distilled water and placed on ice. After the staining solution was removed, the cells were washed twice with JC-1 staining buffer, and 2 mL of DMEM was added. The cells were observed under a confocal laser scanning microscope (Zeiss, Wetzlar, Germany).

### Wound healing assay

The plates were prepared, grouped, and treated with drugs as described above. A yellow pipette tip was used to create a straight vertical scratch on the plate. After scratching, the old culture medium was removed, and the wells were washed twice with PBS. The corresponding concentrations of drugs were added according to the grouping. At 0, 24 and 48 h after scratching, the scratches were photographed under an inverted microscope, and the effects of the drugs on cell migration ability were analyzed.

### Transwell assay

Matrigel hydrogel was diluted with DMEM (1:8), and 50 μL of the diluted hydrogel was added to the upper chamber of the Transwell chamber. Then, 100 μL of a DMEM hydration solution was added to the upper chamber, and 800 μL of DMEM containing 20% FBS was added to the lower chamber. The chamber was placed in an incubator (37°C with 5% CO _2_) and allowed to stand overnight. After 24 or 48 h of drug intervention, the cells were harvested, the old culture medium was removed, and the cells were resuspended in culture medium without FBS to a concentration of 5×10 ^5^ cells/mL.Then, 100 μL of cell suspension was added to the upper chamber of the Transwell chamber, and 600 μL of culture medium containing 20% FBS was added to the lower chamber. After 12 h of incubation, the medium in the upper chamber was removed, and the chamber was washed with PBS and gently wiped with a cotton swab to remove the cells and Matrigel hydrogel from the upper chamber. The chamber was washed twice with PBS and fixed with 4% paraformaldehyde for 15 min. After washing twice with distilled water, the chamber was air-dried, stained with crystal violet staining solution for 10 min, washed with distilled water, and air-dried. The cells that had migrated through the membrane to the lower chamber were observed and photographed under an inverted microscope. The cells were counted, and the effects of the drugs on cell invasion ability were analyzed.

### Animal model construction and drug administration

The experimental animals were SPF-grade female BALB/c nude mice (6–8 weeks old; body weight, 19±1 g), which were purchased from Shanghai SLAC Laboratory Animal Co. (Shanghai, China). The mice were kept in a clean-grade animal laboratory and subjected to a 12/12 h light/dark cycle. The study protocol was ethically reviewed and approved by Shanghai University of Traditional Chinese Medicine (Ethics Permit No. PZSHUTCM211115006).

SK-BR-3 cells in the logarithmic growth phase were trypsinized, and after centrifugation at 300 *g* for 5 min, the supernatant was discarded. The cells were resuspended in PBS and mixed with a Matrigel hydrogel at a ratio of 1:1. Nude mice were injected subcutaneously in the left mammary fat pad with 5×10 ^6^ SK-BR-3 cells. The tumor was measured regularly by using a caliper and tumor volume was calculated with the formula *V* =0.5× *a*× *b* ^2^, where *V* represents tumor volume, *a* represents the maximum tumor diameter, and *b* represents the minimum tumor diameter. After approximately 2 weeks, when the tumor volume reached 100 mm ^3^ or more, drug administration was initiated. XLLXF was administered by gavage at a dose of 5.26 mg/kg once daily. Trastuzumab was administered by intraperitoneal injection at a dose of 5 mg/kg twice a week. The control group was injected with 0.9% saline solution. The tumor volume of each group of tumor-bearing nude mice was measured regularly, and after 4 weeks of continuous drug administration, the mice were euthanized.

### Enzyme-linked immunosorbent assay

After the mice were anaesthetized, blood samples were collected from the orbital vein, transferred to 1.5-mL EP tubes, and allowed to stand at room temperature for 30 min. The samples were centrifuged at 500 *g* for 15 min, and the supernatant was gently collected and transferred to new EP tubes. The tubes were stored at –80°C. An enzyme-linked immunosorbent assay (ELISA) plate (Shanghai Enzyme-linked Biotechnology Co., Ltd., Shanghai China) with immobilized antibodies was obtained, and the diluted standards were added to the first row of wells. Subsequently, 10-fold diluted samples were added to each well, and the plate was incubated for 90 min. The plate was then washed with PBS, and an antibody solution (IL2, IL15, IFN-γ, and TNF-α) was added to each well (except blank wells). After 60 min, the plate was washed with PBS, ABC working solution was added to each well (except for the blank wells), and the plate was incubated for 30 min. After the plate was washed with PBS, a TMB color development solution was added to each well (90 μL per well), and the plate was incubated in the dark for 20 min. The reaction was stopped by adding a TMB stop solution (100 μL per well). The absorbance of each well was measured at 450 nm with an automated microplate reader. The OD values were used to plot a standard curve and calculate sample concentrations.

### Collection of active ingredients in XLLXF and target genes for the treatment of HER2-positive BC

The active ingredients in XLLXF were searched in the TCMSP database. The screening criteria included an oral bioavailability of ≥ 30% and drug-likeness of ≥ 0.18. Relevant literature from databases, such as CNKI and PubMed, was reviewed to confirm and supplement the active ingredients obtained from the TCMSP database. The target proteins of the active ingredients were obtained from the TCMSP database and standardized to standard gene names by using the UniProt database. The gene associated with HER2-positive BC was searched using the keyword “HER2 positive BC” in the GeneCards database. The intersection of the HER2-positive BC-related targets and the targets of XLLXF was determined to identify the active ingredients and targets for the treatment of BC by XLLXF. The results were visualized using a Venn diagram.

### Construction of the “XLLXF-active ingredients-target genes” network

The “XLLXF–active ingredients–target genes” network was visualized using Cytoscape. The Network Analyser tool in Cytoscape was used to calculate degree centrality and select major active ingredients..

### Enrichment analysis and construction of the protein-protein interaction network

Gene Ontology (GO) is widely used in the field of bioinformatics and covers three aspects of biology: cellular component, molecular function, and biological process. The target genes were subjected to GO and KEGG pathway enrichment analyses by using the DAVID database. A protein–protein interaction (PPI) network of the target genes involved in the treatment of HER2-positive BC by XLLXF was constructed using the STRING database. The core proteins with high degree centrality were determined.

### Molecular docking

IL2 (PDB ID: 1IRL), JAK (PDB ID: 6SM8), STAT3 (PDB ID: 6NJS), and TNF (PDB ID: 7KPA) were obtained from RCSB ( https://www.rcsb.org/). Compound structures for curcumenol, icariside-II, lobetyolin, and scutellarein were obtained from the PubChem database ( https://pubchem.ncbi.nlm.nih.gov/). PyMOL 2.3 software was used to remove extraneous small molecules from protein molecules. Then, the protein molecules were imported into AutoDock Tools-1.5.6 software to remove water molecules and add hydrogen atoms and finally saved as pdbqt files. The small molecule compounds were also imported into AutoDock Tools 1.5.6 software to delete water molecules, add atomic charges, assign atom types, and make all flexible bonds rotatable by default and finally saved as pdbqt files. All docking experiments were performed using AutoDock Tools-1.5.6 software ( https://ccsb.scripps.edu/mgltools/downloads/). Molecular docking calculations were performed using the Lamarckian genetic algorithm with the following parameters: population of 150, maximum value of 25 million energy evaluations, maximum number of 2000, crossover rate of 0.8, mutation rate of 0.02, 10 independent docking runs, and final docking structure based on binding free energy. Visualization of docking results was processed using PyMOL 2.3 software.

### Immunohistochemistry (IHC) staining

The slides underwent two rounds of deparaffinization with xylene for 10 min each and rehydrated with ethanol for 10 min. Subsequently, the slides were washed three times with PBS and subjected to antigen retrieval by boiling in a 10 mM sodium citrate buffer solution for 8 min. To eliminate endogenous peroxidase activity, the sections were permeabilized with 3% hydrogen peroxide in methanol at room temperature in the dark. Then, they were blocked with 10% goat serum to minimize nonspecific binding. After three additional washes with PBS, the samples were incubated overnight at 4°C in a humid chamber with primary antibodies diluted at 1:200 (JAK1, clone No. 3H7A8 and TNF-α, clone No. 7B8A11; Proteintech, Wuhan, China). Then, incubation with a 1:200 dilution of HRP-conjugated secondary antibodies (Proteintech, Wuhan, China) was performed. Subsequently, color development was achieved by the addition of 3,3′-diaminobenzidine substrate, and counterstaining was performed using Mayer’s hematoxylin (Absin, Shanghai, China).

### Statistical analysis

GraphPad Prism software was used to generate statistical graphs. Data are presented as the mean±standard deviation. Data analysis was performed using SPSS 25.0 statistical software. One-way analysis of variance was used for intergroup comparisons. LSD or Games-Howell tests were used depending on homogeneity of variance. A *P* value less than 0.05 was considered statistically significant.

## Results

### XLLXF synergizes with trastuzumab to inhibit proliferation in HER2-positive BC cells

The chemical components of XLLXF were identified using UPLC-Q-TOF/MS, and the total ion chromatograms of XLLXF in positive and negative ion modes are shown in Supplementary Figure S1. Through the establishment of a self-built chemical component database and literature analysis, the chemical constituents of XLLXF were comprehensively identified according to accurate molecular weights and retention times. Ultimately, 65 chemical components were identified in XLLXF: 33 flavonoids, 6 terpenes, 4 phenolic acids, 4 alkaloids, 4 fatty acids, 3 amino acids, 3 sugars, and 8 other components ( Supplementary Table S1). The main chemical constituents of XLLXF include icariin, sophoricoside, ginsenoside Rb1, curcumin, resveratrol, baicalin, luteolin, and baicalein.

**Figure 1:**
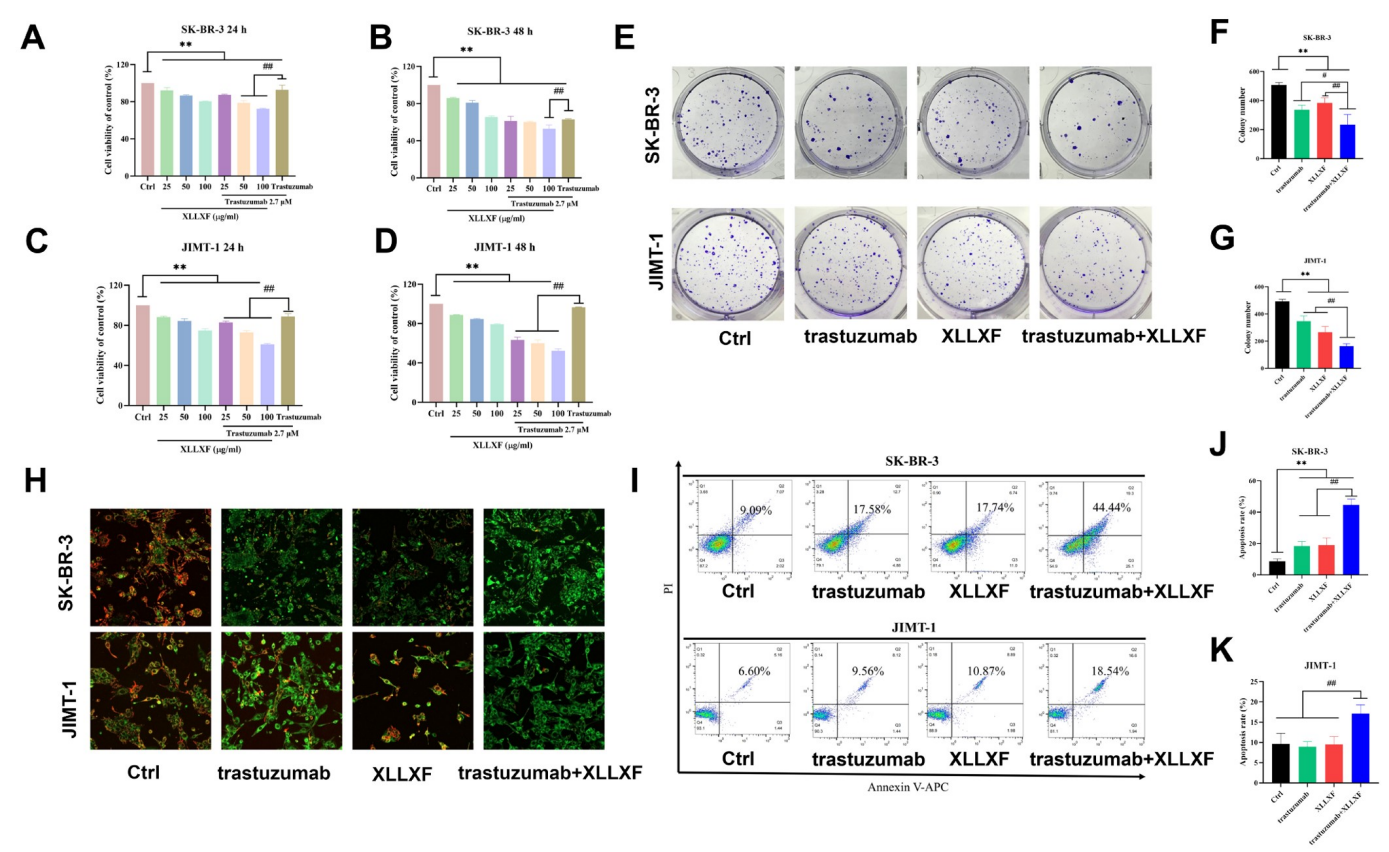
Figure 1 XLLXF combined with trastuzumab inhibits viability of HER2-positive BC cells (A,B) Effects of intervention for 24 and 48 h on the proliferation ability of SK-BR-3 cells. (C,D) Effects of intervention for 24 and 48 h on the proliferation ability of JIMT-1 cells. (E–G) Effects of intervention for 24 h on the colony-forming ability of SK-BR-3 and JIMT-1 cells. (H) JC-1 experiment assessing the impact of the drugs on the mitochondrial membrane potential of BC cells; green fluorescence indicates early apoptotic cells. (I–K) Flow cytometry analysis examining the effects of XLLXF intervention for 24 h on the apoptosis of SK-BR-3 and JIMT-1 cells. *P<0.05 vs control group; **P<0.01 vs control group; # P<0.05 vs trastuzumab+XLLXF group; ##P<0.01 vs trastuzumab+XLLXF group.

To evaluate the effects of XLLXF on HER2-positive BC cells, we treated SK-BR-3 and JIMT-1 cells with different concentrations of XLLXF (25, 50, and 100 μg/mL) alone or in combination with trastuzumab (2.7 μM). We also included a blank control group and a trastuzumab group (2.7 μM) for comparison. The results showed that XLLXF dose-dependently inhibited the proliferation of SK-BR-3 and JIMT-1 cells after 24 and 48 h of treatment. Compared with trastuzumab alone, the combination of XLLXF and trastuzumab enhanced the inhibitory effect on BC cell proliferation. For SK-BR-3 cells, XLLXF at different concentrations significantly inhibited cell proliferation after 24 and 48 h of treatment, and this effect showed a clear dose dependency ( *P*<0.01). Compared with trastuzumab alone, the combination of XLLXF and trastuzumab at different concentrations achieved good inhibitory effects on cell proliferation after 24 h of treatment ( *P*<0.01; Figure 1A). After 48 h, the combination of 100 μg/mL XLLXF and trastuzumab achieved a 52.70% inhibition rate, which was statistically significant compared with that of the trastuzumab-alone group ( *P*<0.01; Figure 1B). For JIMT-1 cells, XLLXF at different concentrations significantly inhibited cell proliferation after 24 and 48 h of treatment, and this effect showed a clear dose dependency ( *P* <0.01; Figure 1C,D). Trastuzumab alone had no significant inhibitory effect on cell proliferation, but when combined with XLLXF, it showed dose-dependent inhibitory effects after 24 and 48 h of treatment ( *P*<0.01).

### XLLXF synergizes with trastuzumab to inhibit colony-forming ability of HER2-positive BC cells

We performed colony formation assays to evaluate the effects of XLLXF combined with trastuzumab on the colony formation ability of SK-BR-3 and JIMT-1 cells. After approximately 2 weeks, the number of colonies was counted. The results showed that compared with the control group, the number of colonies in the trastuzumab group, XLLXF group, and trastuzumab+XLLXF group decreased significantly for SK-BR-3 cells ( *P*<0.01; Figure 1E,F). The same trend was observed for JIMT-1 cells ( *P*<0.01; Figure 1E,G). Moreover, compared with the single-drug groups, the combination of trastuzumab and XLLXF showed the strongest inhibitory effect on colony formation ability ( *P*<0.05, *P*<0.01).

### XLLXF synergizes with trastuzumab to promote apoptosis in HER2-positive BC cells

To investigate the effects of XLLXF combined with trastuzumab on the apoptosis of HER2-positive BC cells, we measured the apoptosis levels of SK-BR-3 and JIMT-1 cells. A decrease in mitochondrial membrane potential is an important feature of early apoptosis. When the mitochondrial membrane potential is high, JC-1 accumulates in the mitochondrial matrix, forming aggregates with red fluorescence. When the mitochondrial membrane potential is low, JC-1 does not accumulate in the mitochondrial matrix, resulting in monomers with green fluorescence. The results showed that compared with the control group, green fluorescence intensity increased, and red fluorescence intensity decreased in all treatment groups, indicating a decrease in mitochondrial membrane potential. The combination of trastuzumab and XLLXF showed the best effect ( Figure 1H).

Flow cytometry analysis showed that after 24 h of treatment, the apoptosis rates of SK-BR-3 cells in the control, trastuzumab, XLLXF, and trastuzumab+XLLXF groups were 8.72%, 18.40%, 18.99%, and 44.73%, respectively. The apoptosis rates of all treatment groups were significantly higher than the apoptosis rate of the control group ( *P*<0.01). Compared with the single-drug groups, the combination of trastuzumab and XLLXF significantly increased the apoptosis level of SK-BR-3 cells ( *P*<0.01; Figure 1I,J). Similar results were observed in the JIMT-1 cells, and the combination of trastuzumab and XLLXF showed a significant increase in apoptosis compared with the other three groups ( *P*<0.01; Figure 1I,K).

### XLLXF synergizes with trastuzumab to inhibit the migration ability in HER2-positive BC cells

**Figure 2:**
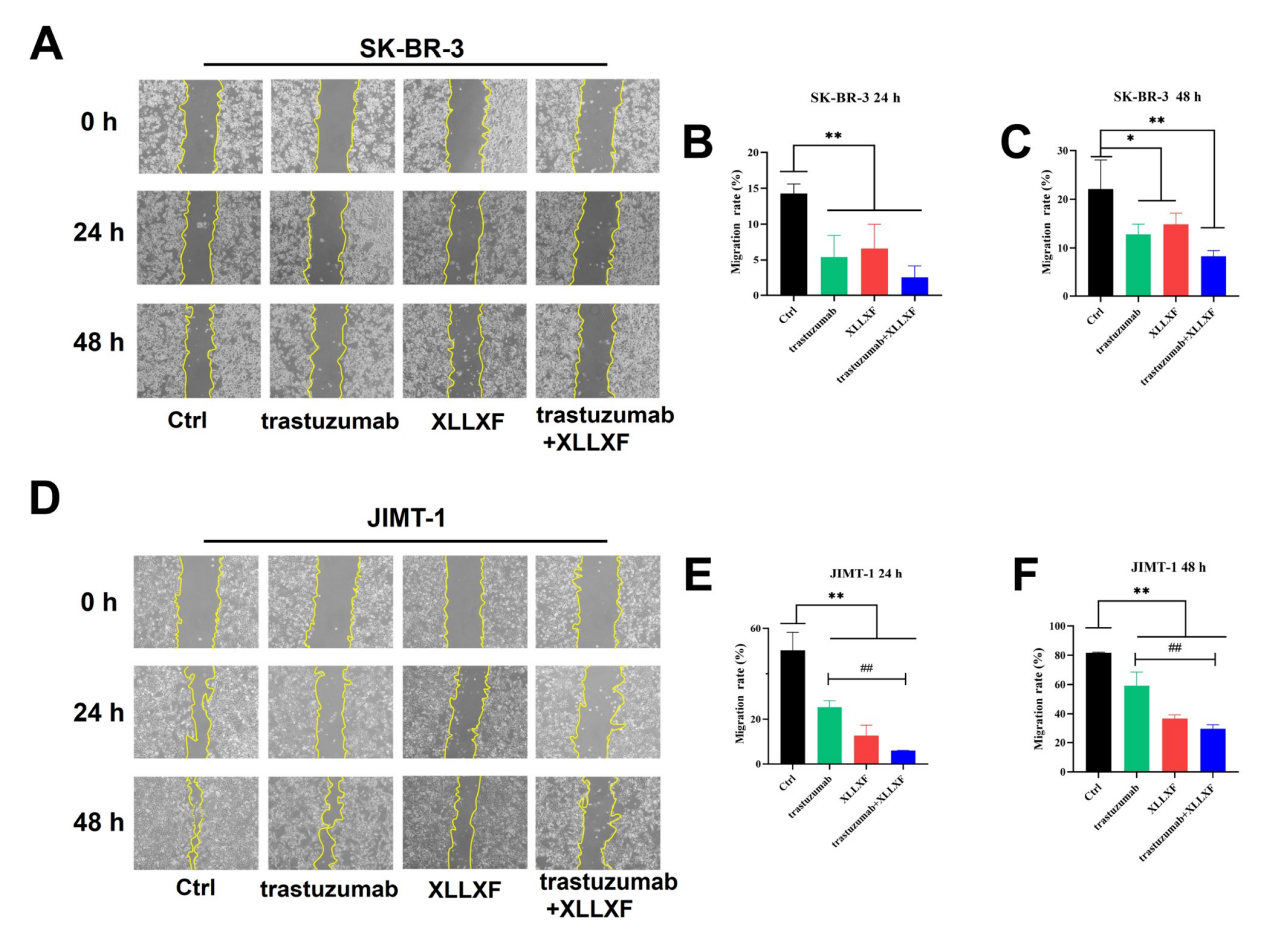
Figure 2 XLLXF combined with trastuzumab inhibits the migration ability of HER2-positive BC cells (A–C) Results and corresponding statistical analysis of the wound healing assay after drug intervention for 24 and 48 h in SK-BR-3 cells. (D–F) Results and corresponding statistical analysis of the wound healing assay after drug intervention for 24 and 48 h in JIMT-1 cells. *P<0.05 vs control group; **P<0.01 vs control group; ## P<0.01 vs trastuzumab+XLLXF group.

To investigate the effects of XLLXF combined with trastuzumab on the migration ability of HER2-positive BC cells, we performed wound healing assays. Compared with the control group, the trastuzumab, XLLXF, and trastuzumab+XLLXF groups showed significantly inhibited migration ability of SK-BR-3 cells after 24 and 48 h of treatment ( *P*<0.05, *P*<0.01; Figure 2A–C). Similar results were obtained for JIMT-1 cells, and the combination of trastuzumab and XLLXF showed a significant inhibitory effect on cell migration compared with trastuzumab alone ( *P*<0.01; Figure 2 D–F).

### XLLXF synergizes with trastuzumab to inhibit the invasion ability in HER2-positive BC cells

**Figure 3:**
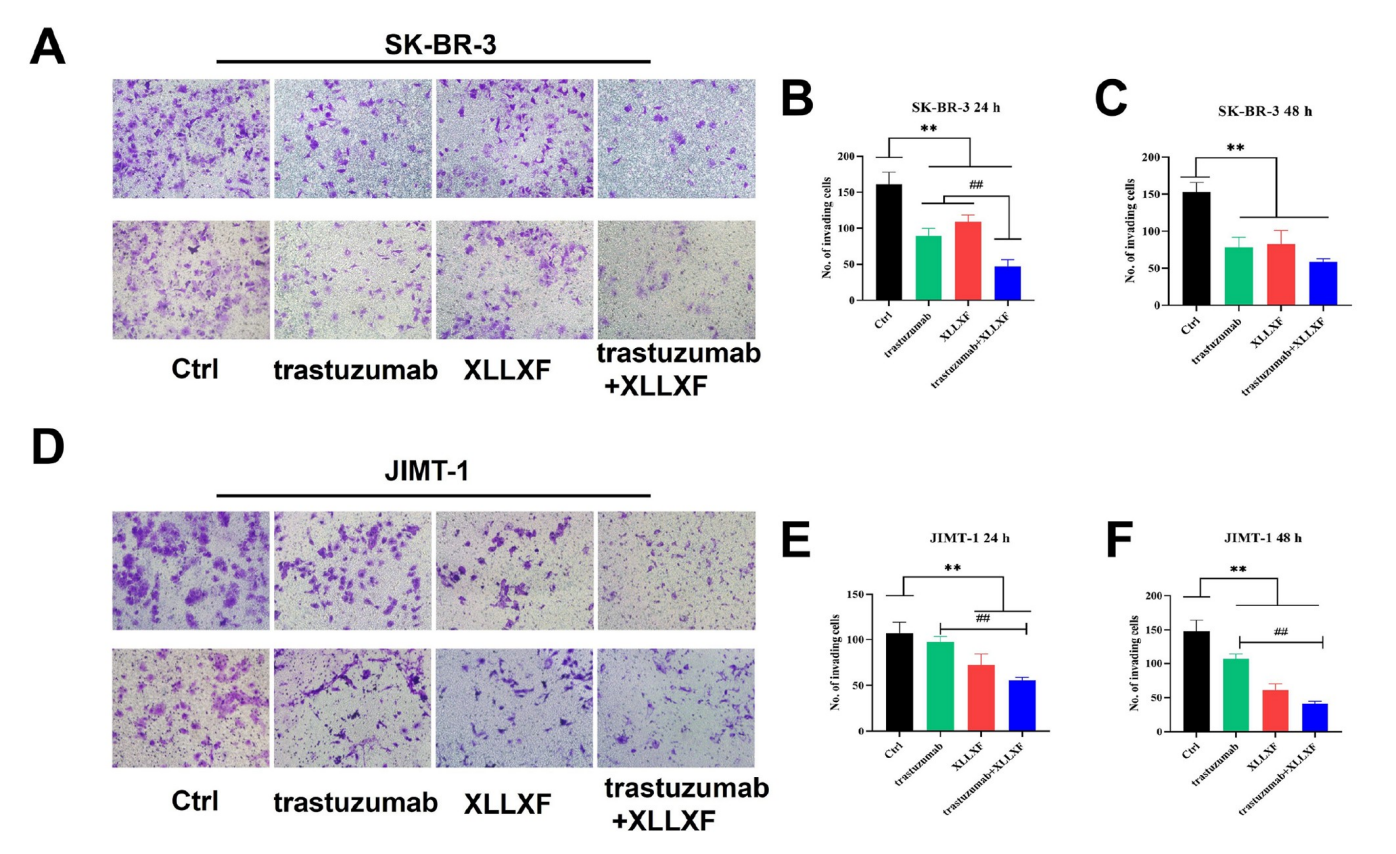
Figure 3 XLLXF combined with trastuzumab inhibits the invasive ability of HER2-positive BC cells (A–C) Results and corresponding statistical analysis of the invasion assay after drug intervention for 24 and 48 h in SK-BR-3 cells. (D–F) Results and corresponding statistical analysis of the invasion assay after drug intervention for 24 and 48 h in JIMT-1 cells. **P<0.01 vs control group; ## P<0.01 vs trastuzumab+XLLXF group.

Transwell assays were performed to evaluate the effects of XLLXF combined with trastuzumab on the invasion ability of HER2-positive BC cells. The results showed that trastuzumab, XLLXF, and trastuzumab+XLLXF significantly inhibited the invasion ability of SK-BR-3 cells. After 24 and 48 h of treatment, the combination of trastuzumab and XLLXF showed a more significant inhibitory effect on the invasion ability of SK-BR-3 cells than the single-drug groups ( *P*<0.01; Figure 3A–C). Similar results were obtained in JIMT-1 cells, and the combination of trastuzumab and XLLXF showed a more significant inhibitory effect on cell migration than trastuzumab alone ( *P*<0.01; Figure 3D–F).

### Active ingredients and target proteins for the treatment of XLLXF in HER2-positive BC

**Figure 4:**
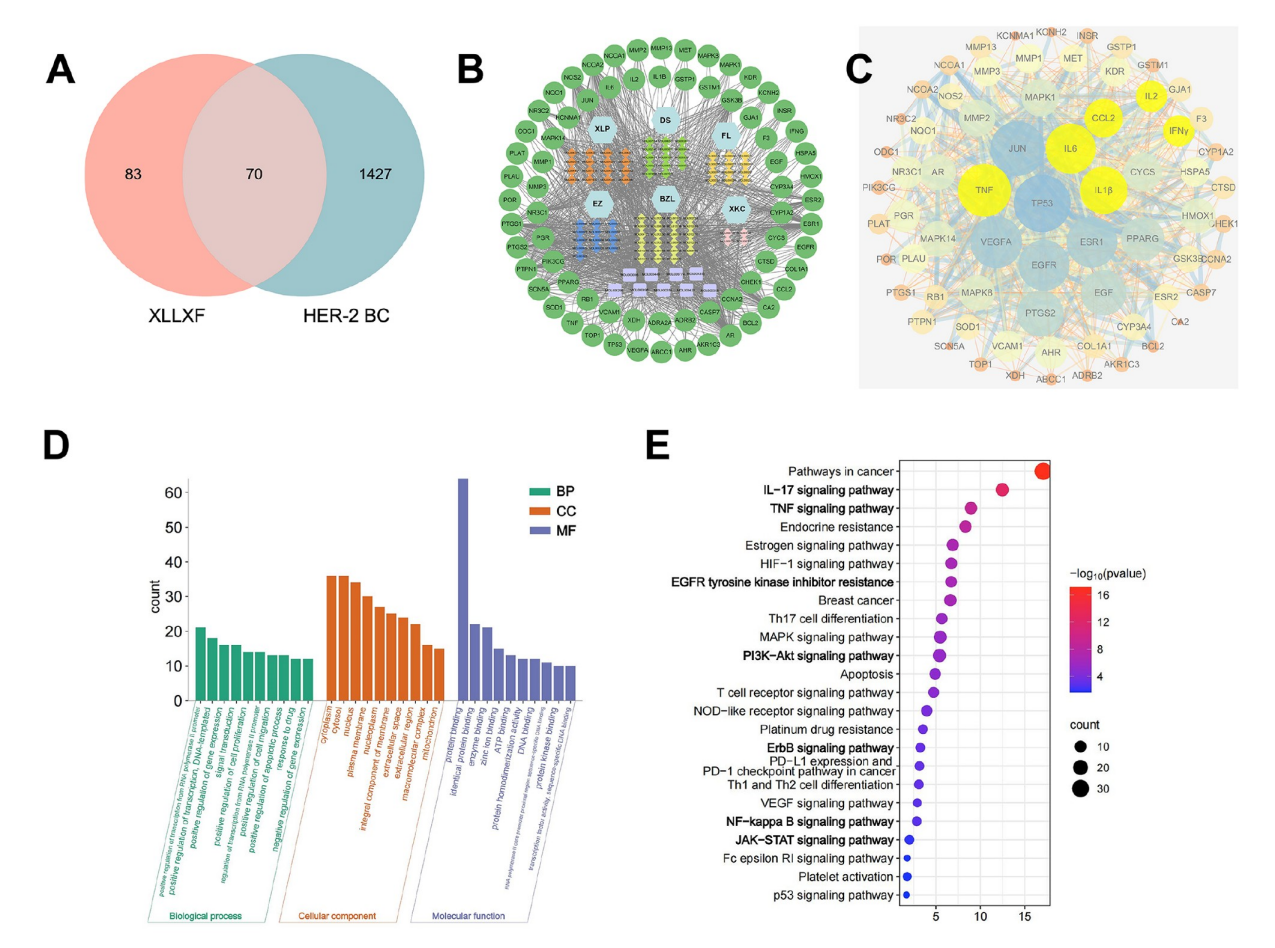
Figure 4 Potential targets of XLLXF in the treatment of HER2-positive BC (A) Intersection targets of XLLXF and HER2-positive BC. (B) The “XLLXF-active components-targets interaction” network. (C) PPI network diagram of intersection targets. (D) Biological processes, cellular components, and molecular functions involved in intersection targets. (E) Pathway enrichment of intersection targets.

We identified the active ingredients of XLLXF and the target proteins corresponding to the active ingredients of XLLXF by screening the TCMSP database. The gene names of the target proteins were standardized using the UniProt database, resulting in a total of 154 gene targets associated with XLLXF. Further search in the GeneCards database identified 1497 genes related to HER2-positive BC. The intersection of these gene sets revealed 70 common targets ( Figure 4A). These ingredients have been reported to have antitumor effects and play a role in the treatment of HER2-positive BC. The active ingredients and target proteins obtained were imported into Cytoscape software to construct an “XLLXF–active ingredients–target proteins” network diagram ( Figure 4B). By using Network Analyser, we calculated the degree to identify top-ranking active ingredients, which included quercetin, luteolin, kaempferol, daucosterol, β-sitosterol, wogonin, baicalein, icaritin, hederagenin, and resveratrol.

### Potential targets of XLLXF for the treatment of HER2-positive BC

By analyzing the PPIs of the potential targets with the STRING database, we identified several highly interactive targets, including TP53, VEGFA, epidermal growth factor receptor (EGFR), IL6, IL1β, PTGS2, EGF, PPARG, CCL2, mitogen-activated protein kinase (MAPK) 1, MMP2, MAPK14, MAPK8, HMOX1, NR3C1, and IL2 ( Figure 4C). EGFR is a member of the HER family, and the MAPK family, including MAPK1, MAPK8, MAPK14, and PIK3CG, is closely related to the antitumor mechanism of trastuzumab [ 4, 9]. The interactions of the transmembrane glycoproteins CD27 and CD70 with members of the TNF superfamily on natural killer (NK) cells can induce the release of perforin, exerting an antitumor effect [10]. The relationships of factors, such as IL-6, CCL2, and IL1β, with NK cell regulation have been reported [ 11, 12] (highlighted in yellow).

### Enrichment analysis

Enrichment analysis of the 70 potential targets revealed their involvement in various biological processes, cellular components, and molecular functions. The biological processes included the positive regulation of RNA polymerase II promoter transcription, positive regulation of transcription, signal transduction, and drug response. The cellular components included cytoplasm, cytosol, nucleus, plasma membrane, extracellular space, and mitochondria ( Figure 4D). The molecular functions included protein binding, enzyme binding, zinc ion binding, and transcription factor activity. KEGG pathway analysis identified 131 pathways, including EGFR tyrosine kinase inhibitor resistance and ErbB, PI3K-Akt, MAPK, JAK-STAT, NF-κB, TNF, T-cell receptor, FcεRI, IL-17, and HIF-1 signaling pathways ( Figure 4E).

### XLLXF increases the antitumor effect of trastuzumab

**Figure 5:**
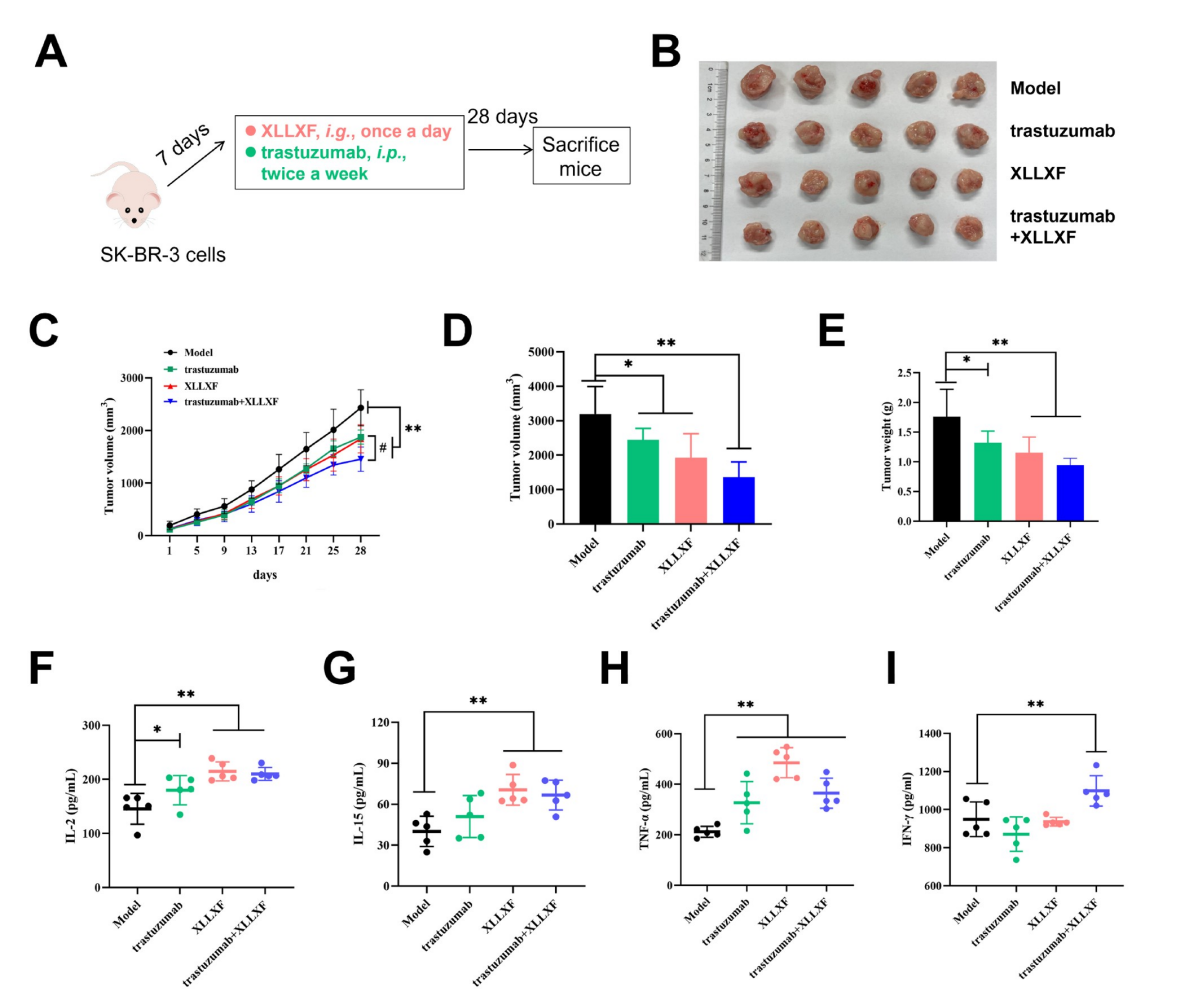
Figure 5 XLLXF increases the antitumor effect of trastuzumab and the combination regulates the expressions of cytokines in tumor-bearing nude mice Schematic diagram of the modelling and administration process; model group: i.g., saline, once daily; trastuzumab group: i.p., 5 mg/kg, twice a week; XLLXF group: i.g., 5.26 mg/kg, once daily; trastuzumab+XLLXF group: trastuzumab, i.p., 5 mg/kg, twice a week, and XLLXF, i.g., 5.26 mg/kg, once daily. (B) Macroscopic observation of tumors in each group. (C) Tumor growth trends in each group. (D) Tumor volume. (E) Tumor weight. (F–I) Expressions of IL2, IL-15, TNF-α, and IFN-γ. *P<0.05 vs control group; **P<0.01 vs control group; #P<0.05 vs trastuzumab+XLLXF group.

We investigated whether XLLXF combined with trastuzumab has the same effects on HER2-positive BC xenografts in nude mice. We established a tumor-bearing nude mouse model by injecting SK-BR-3 cells into mammary fat pads. The mice were treated with XLLXF at a dose of 5.26 mg/kg by oral gavage once a day and trastuzumab at a dose of 5 mg/kg by intraperitoneal injection twice a week ( Figure 5A). After 4 weeks of treatment, the tumors were measured, and the results showed that XLLXF combined with trastuzumab significantly inhibited tumor growth compared to the control group ( Figure 5B–E).

### XLLXF in combination with trastuzumab regulates the expressions of cytokines

The concentrations of IL-2, IL-15, TNF-α, and IFN-γ in the sera were measured in the control, trastuzumab, XLLXF, and trastuzumab+XLLXF groups. The results showed that XLLXF combined with trastuzumab significantly increased the levels of IL-15, IL-2, TNF-α, and IFN-γ in the sera of tumor-bearing nude mice ( Figure 5F–I). These findings suggested that XLLXF combined with trastuzumab can enhance the activation of NK cells and the secretion of cytokines involved in antitumor immune responses.

### Molecular docking analysis

**Figure 6:**
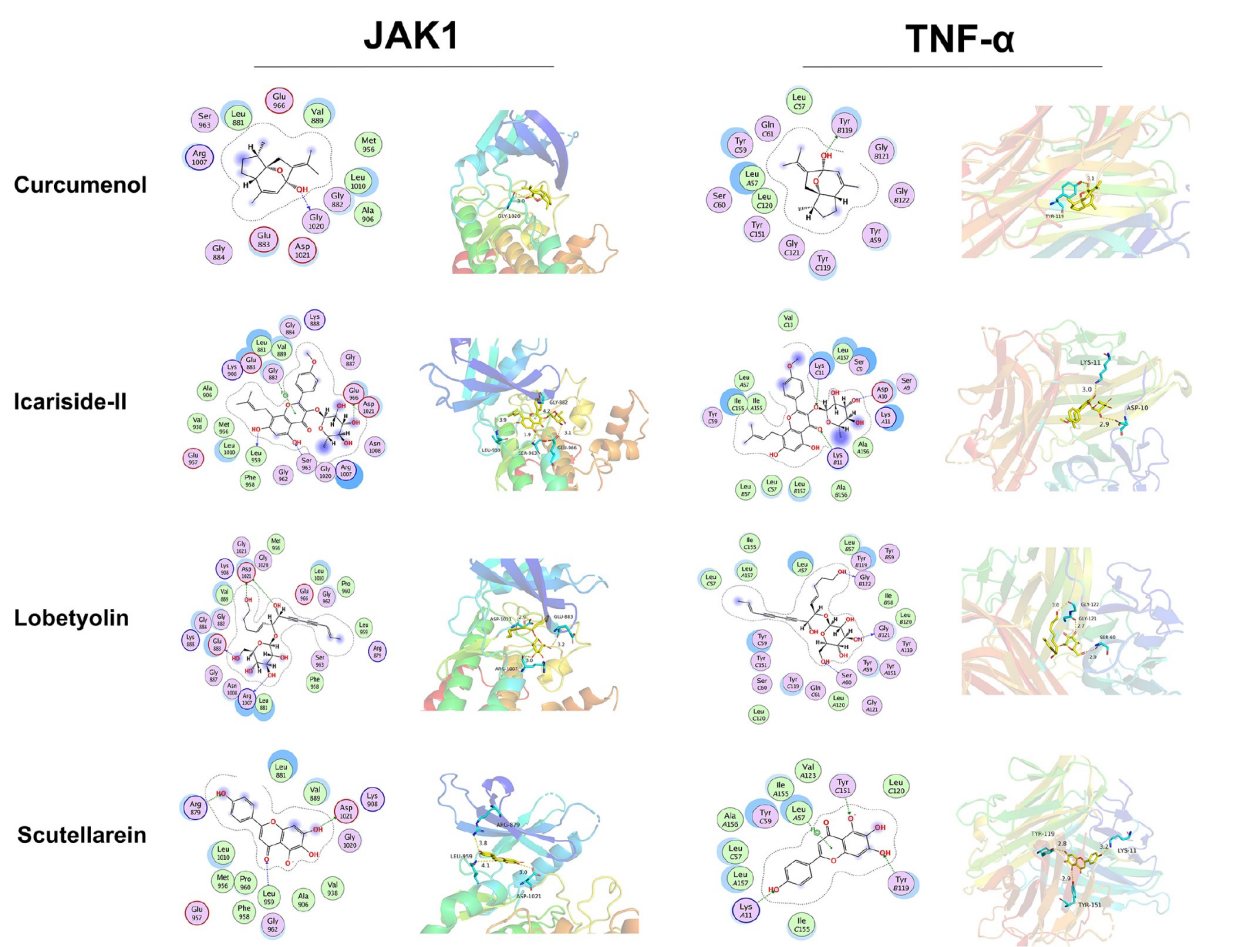
Figure 6 Schematic representation of the binding modes between the four compounds of XLLXF and JAK1 and TNF-α

Four immune-related targets (IL2, JAK, STAT, and TNF) were selected and docked with four compounds, curcumenol, icariside-II, lobetyolin, and scutellarein. The results showed that JAK and TNF had high binding affinity with all four compounds ( Supplementary Table S2). For example, icariside-II interacts with the active site of the JAK protein (3.0 Å) and interacts with amino acid residues, such as ALA-906, VAL-938, MET-956, LEU-1010, LEU-959, PHE-958, SER-963, GLU-966, and GLY-882. Icariside-II can form strong hydrogen bond interactions with amino acids (LEU-959, SER-963, and GLU-966) at the active site, indicating strong binding and an important role in anchoring molecules in the protein cavity. Additionally, this compound exhibits strong hydrophobic interactions with hydrophobic amino acids (ALA-906, VAL-938, MET-956, LEU-1010, LEU-959, and PHE-958) in the protein cavity. The phenyl ring of the compound can form pi-H conjugation interactions with GLY-882, contributing to the stability of small molecules in the cavity. Lobetyolin exhibits good complementarity with the active cavity of the TNF protein, forming strong hydrogen bond interactions with amino acids, such as GLY-122, GLY-121, and SER-60, which plays an important role in anchoring small molecules in the protein pocket. Furthermore, this compound displays strong hydrophobic interactions with hydrophobic amino acids, such as ILR-58, LEU-120, LEU-57, LEU-157, ILE-155, and LEU-120, greatly contributing to the stabilization of small molecules in the pocket ( Figure 6).

### XLLXF in combination with trastuzumab regulates the protein expressions of JAK1 and TNF-α in tumor-bearing nude mice

**Figure 7:**
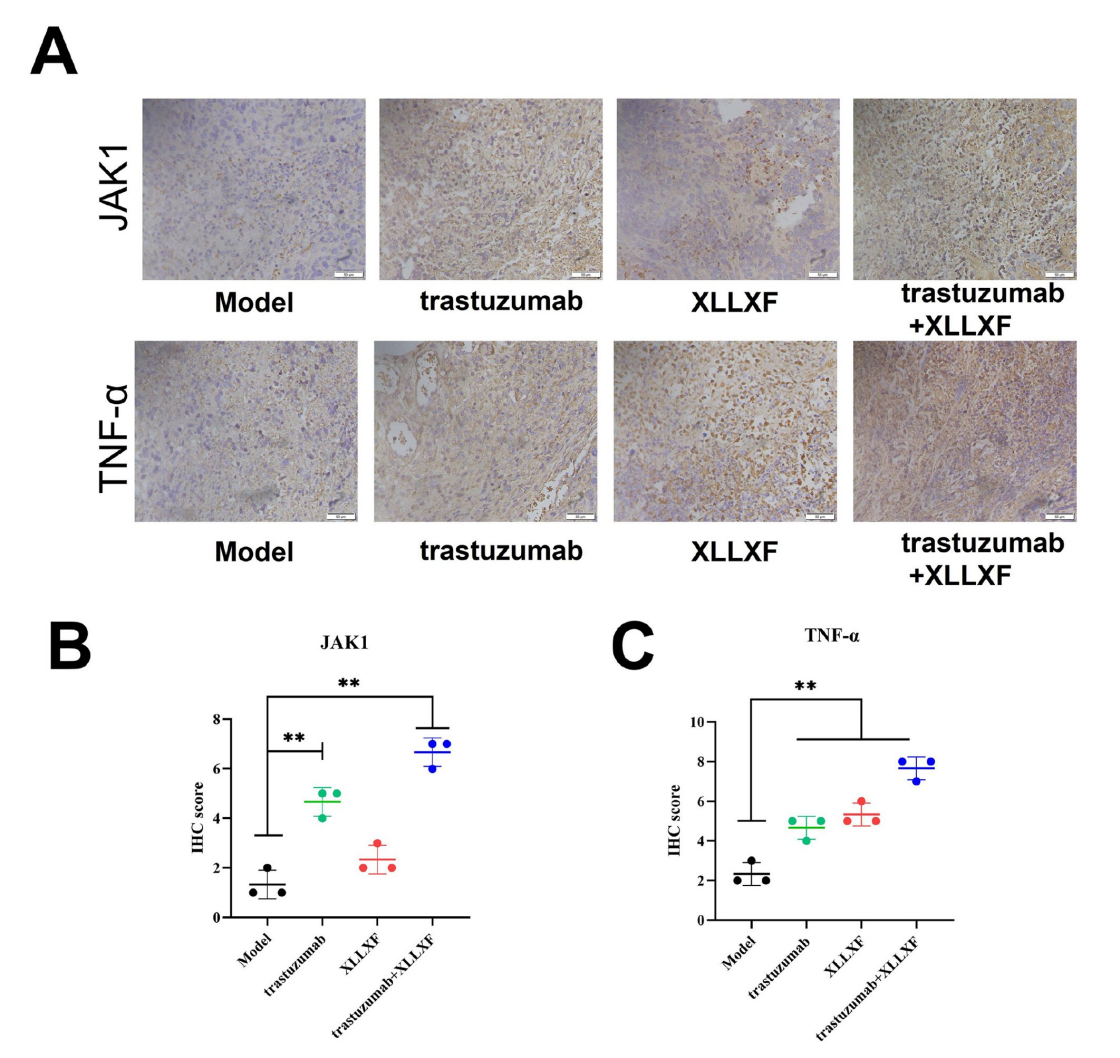
Figure 7 XLLXF in combination with trastuzumab regulates the protein expressions of JAK1 and TNF-α in tumor-bearing nude mice (A) Protein expressions of JAK1 and TNF-α in different groups revealed by IHC. (B,C) The statistical results of IIHC. **P<0.01 vs model group.

Referencing the results of molecular docking, we hypothesized that XLLXF may play an immunomodulatory role in HER2-positive BC by regulating the expressions of JAK and TNF. In the XLLXF combined with trastuzumab groups, we observed increased expressions of JAK1 and TNF-α on lymphocytes in tumor tissues by IHC staining ( Figure 7).

## Discussion

HER2, a member of the EGFR family, plays a pivotal role in HER2-positive BC [ 13, 14]. Before the development of targeted therapies, the primary approach for adjuvant treatment of HER2-positive BC relies on chemotherapy. Trastuzumab ushered in a new era of targeted therapy for HER2-positive BC. By binding to HER2, trastuzumab reduces the formation of homodimers and impedes growth signal transduction, effectively inhibiting the progression of BC [15]. However, some patients do not show long-term benefits from trastuzumab therapy [16].

Network pharmacology has gained popularity in BC research. It involves the systematic analysis of drug–target interactions and exploration of complex networks within biological systems [17]. By integrating multiomics data and network analysis, researchers can identify crucial molecular pathways and key nodes involved in BC development. This knowledge provides valuable insights into the underlying mechanisms of the disease and facilitates the discovery of novel therapeutic strategies [18]. In this study, we explored the potential target of XLLXF to inhibit HER2-positive BC by network pharmacology. We focused on inflammation-related targets, such as JAK, TNF, IL2, and IL15.

The growth and prognosis of tumors are closely associated with the expressions of inflammatory cytokines [19]. IL2 and IL15 are two well-known cytokines with immune-activating functions [ 20, 21]. They can stimulate and sustain the activity of CD8 ^+^ T cells and NK cells, enhancing their ability to lyse tumors [22]. Currently, IL2 and IL15 have been widely utilized in cellular engineering [ 23, 24], preclinical trials, and phase I trials [ 25, 26]. IFN-γ, a proinflammatory factor, is secreted by activated T cells and NK cells, and plays a crucial role in both systemic and local immunity. Through the regulation of signaling pathways such as JAK/STAT and Fas/FasL, IFN-γ can orchestrate diverse immune responses, participating in tumor growth inhibition and tumor immune evasion. It plays a vital role in the immunological development of tumors [ 27, 28]. A clinical study has shown that patients with HER2-positive BC and on neoadjuvant therapy have a significantly increased number of activated NK cells compared with patients who achieved pathological complete response [29] [29]. TNFα-TNFR2 signaling upregulates the expressions of CD25 (IL-2Rα) and nutrient transport proteins in NK cells, inducing a metabolic shift toward aerobic glycolysis and promoting the proliferation of NK cells [30]. These findings suggested that the inhibitory effects of cytokines on tumors are mediated through the enhancement of tumor-killing cells.

Currently, research into the regulation of inflammation by TCM and its compounds is being conducted progressively. For instance, the *Jiawei-Yanghe* decoction has been shown to reduce the expression levels of IL-1β, IL-6, TNFα, PTGS2, and VEGFα in BC tissues, and thereby decrease the expressions of M2 macrophages and Treg cells with increased expressions of M1 macrophages [31]. Norcantharidin, an effective IL-6 inhibitor, enhances the antitumor activity of sorafenib in a rat model of hepatocellular carcinoma through the IL-6–STAT3 pathway [32]. The classic TCM formula *Si-Wu* decoction significantly decreases the levels of IL-6, TNF-α, and IL-1β, effectively treating ulcerative colitis [33]. In this study, we found that XLLXF exhibits inhibitory effects on the malignant biological behavior of HER2-positive BC cells treated with trastuzumab and can regulate the secretion of cytokines. Referencing these findings, we speculate that XLLXF may enhance tumor immune capability by increasing the expression of cytokines.

In summary, this study provides an effective synergistic approach for targeted therapy in HER2-positive BC. TCM plays an important role in the complex tumor immune microenvironment because of its multitarget and multipathway features. However, elucidating the regulatory mechanism of this cascade is difficult, and technical approaches and validation are needed to deeply dissect the effects of XLLXF-regulated cytokine expression in HER2-positive BC.

## Supplementary Data

Supplementary data is available at *Acta Biochimica et Biophysica Sinica* online.
